# Time and risk preferences and the perceived effectiveness of incentives to comply with diabetic retinopathy screening among older adults with type 2 diabetes

**DOI:** 10.3389/fpsyg.2023.1101909

**Published:** 2023-04-17

**Authors:** Jianjun Tang, Ziwei Yang, Frank Kee, Nathan Congdon

**Affiliations:** ^1^School of Agricultural Economics and Rural Development, Renmin University of China, Beijing, China; ^2^College of Economics and Management, Huazhong Agricultural University, Wuhan, China; ^3^Centre for Public Health, Queen's University Belfast, Belfast, United Kingdom; ^4^Orbis International, New York, NY, United States; ^5^Zhongshan Ophthalmic Center, Sun Yat-sen University, Guangzhou, China

**Keywords:** economic experiments, screening compliance, behavioral economics, persons with diabetes, China

## Abstract

Behavioral economics has the potential to inform the design of incentives to improve disease screening programs by accounting for various behavioral biases. We investigate the association between multiple behavioral economics concepts and the perceived effectiveness of incentive strategies for behavioral change among older patients with a chronic disease. This association is examined by focusing on diabetic retinopathy screening, which is recommended but very variably followed by persons living with diabetes. Five time and risk preference concepts (i.e., utility curvature, probability weighting, loss aversion, discount rate, and present-bias) are estimated simultaneously in a structural econometric framework, based on a series of deliberately-designed economic experiments offering real money. We find that higher discount rates and loss aversion and lower probability weighting are significantly associated with lower perceived effectiveness of intervention strategies whereas present-bias and utility curvature have an insignificant association with it. Finally, we also observe strong urban vs. rural heterogeneity in the association between our behavioral economic concepts and the perceived effectiveness of intervention strategies.

## 1. Introduction

Diabetes is an increasingly pressing global health care problem, with 537 million people living with diabetes worldwide in 2021, estimated to increase to 783 million by 2045 (Sun et al., 2022). The disease is associated with an increased risk of all-cause mortality from a range of cardiovascular and non-cardiovascular diseases, such as acute myocardial infarction, stroke, kidney or heart failure, and can also lead to blindness (Chatterjee et al., 2017; Rawshani et al., 2018). Diabetes in adults creates a tremendous global economic burden of US$1.31 trillion, 1.8% of the total global gross domestic product (Bommer et al., 2017). China, in particular, has experienced one of the most dramatic rises in diabetes prevalence, which increased by more than twelve-fold from 0.87% in 1980 to 10.9% in 2013 (Wang et al., 2017). This amounts to 110 million people in the country living with diabetes (Ma, 2018), inflicting upon China the world’s largest diabetes epidemic.

Diabetic retinopathy (DR), a primary retinal vascular complication of diabetes, is the leading cause of blindness among working-age adults around the world. A systematic review has shown that the prevalence of DR among people living with diabetes just exceeds 18% in China (Song et al., 2018), implying that some 20.3 million people in the country live with DR. Early-stage DR is generally asymptomatic and almost entirely preventable, however untreated DR can cause severe and irreversible vision impairment and ultimately progress to blindness. Consequently, timely screening for DR is vital in the management of those with diabetes. There is reliable evidence suggesting that annual screening is cost-effective, and screening is recommended in many current guidelines. However, 56.8% of persons with diabetes in China have never had an eye examination and only one-third have received eye examinations in the previous year (Wang et al., 2010). A more recent study in Shanghai reports a higher rate of examination (41.9%), but one which still falls far short of recommendations (Zhu et al., 2020).

Behavioral economics, which occupies the intersection between economics and behavioral sciences, has increasingly focused on studying individual-level health behavior (Matjasko et al., 2016). In particular, behavioral economic concepts are reported to be associated with diabetic self-management, providing the potential to design interventions to incentivize healthier lifestyles. For instance, a systematic review shows that time preference, which refers to the extent to which future benefits are preferred to present gratification, is repeatedly found to be associated with self-management among persons with diabetes (Madsen et al., 2019). Additionally, risk-aversion has also been found to be associated with better diabetic self-management (Simon-Tuval et al., 2016).

Several previous studies have attempted to use incentives to improve self-management among people with diabetes (Tambor et al., 2016; Judah et al., 2018), but we know little about how time and risk preferences are associated with older patients’ perceptions of incentive strategies for behavioral change. We need to better understand the mechanism behind these decision-making processes, so that we can design better incentive-based interventions customized to individual-specific decision-making phenotypes. In the present study, we address these knowledge gaps by investigating the association between behavioral economic traits and the perceived effectiveness of intervention strategies to enhance patient compliance with retinal screening.

Our objectives are two-fold. Firstly, we focus on multiple behavioral economic concepts simultaneously, filling a gap left by many previous studies which have tended to concentrate on a single trait. We base our measurements of time and risk preferences on deliberately-conducted economic experiments offering real money, and estimate these concepts simultaneously in a structural econometric framework. Second, to the best of our knowledge no study has previously investigated the impacts of behavioral biases on willingness to comply with DR screening and the perceived effectiveness of various incentive strategies to promote compliance.

The remainder of the paper is structured as follows. Section 2 presents the conceptual framework and literature review. Section 3 deals with experimental design, and section 4 presents data collection and descriptive characteristics. Section 5 displays the empirical results and section 6 concludes and discusses policy implications.

## 2. Conceptual framework and literature review

Perceived effectiveness of intervention strategies refers to the extent to which a specific intervention strategy is perceived as likely to be effective for the targeted behavioral change (Bain et al., 2020) and could inform the design of interventions tailored to the attitudes of older patients to improve the uptake of DR exams (Yan et al., 2012; Chen et al., 2020). Utilizing this notion allows us to examine how readily a patient with diabetes might comply with the recommended DR screening. Insights from behavioral economics are being increasingly used to better understand the decision-making processes of diabetes self-management. We consider five behavioral economic concepts, namely discount rates, present-bias, risk preference, probability weighting, and loss aversion. Based on a brief literature review, we discuss the potential influencing mechanism of each of the five behavioral economic concepts on participants’ perceived effectiveness of intervention strategies.

### 2.1. Discount rates

Discount rates refer to the extent to which immediate gratifications are preferred over future health benefits (O'Donnell et al., 2021). A systematic review demonstrated that patients with diabetes exhibiting higher discount rates have poorer glycemic control and outcomes than those with lower discount rates (Madsen et al., 2019). Time preference is associated with poorly-controlled HbA1c levels (Lebeau et al., 2016; Lansing et al., 2017; Stoianova et al., 2018; Reach et al., 2018a; Epstein et al., 2019), non-adherence to medication (Reach et al., 2018a,b), poor self-management (Karl et al., 2018; Shain et al., 2022), and depression (Campbell and Egede, 2022) among persons with diabetes.

Existing studies have consistently reported that people with lower discount rates, that is those who are more ready to delay immediate gratification, are more likely to undergo cancer screening (Bradford et al., 2010; Goldzahl, 2017) or to adhere to surveillance for asymptomatic disease (Kim and Radoias, 2016). Routine DR screening does not lower the risk of developing DR but rather can detect it at an earlier, more treatable stage. People with potential health risks face the intertemporal choice either to make immediate efforts at detecting and treating disease in its early stage, or to procrastinate and forego earlier detection of disease which might otherwise deteriorate to a worse future stage. Thus, persons with diabetes and lower discount rates are expected to be more likely to undergo DR screening to avoid future vision loss and associated costs.

### 2.2. Present-bias

A similar yet distinct concept is present-bias, that is, favoring long-term health benefits but choosing to enjoy immediate gratification. It is also conceptualized as self-control problems, which means that present-biased people are patient when making future plans but do not stick to the plans when an immediate decision needs to be made (O'Donoghue and Rabin, 1999, 2015). Although extensive studies have established its association with unhealthy behaviors (Kang and Ikeda, 2016; Hunter et al., 2018; Stoklosa et al., 2018; Wang and Sloan, 2018), we are aware of only two studies that have investigated the role of present-bias in self-management behaviors among patients with diabetes. Mørkbak et al. (2017) showed that present-bias is related to the onset and self-management of diabetes as well as its health outcomes. Similarly, based on the Health and Retirement Study dataset in the United States, Wang and Sloan (2018) observed that present-biased individuals with diabetes have reduced probabilities of following diabetic care guidelines. DR screening detects disease in its early stage and brings about potential future benefits, however, it requires individuals to make immediate financial, time, and psychological efforts. Therefore, present-biased individuals might care about their future health but are expected to forego earlier detection of disease due to their self-control problems.

### 2.3. Utility curvature

Utility curvature, which refers to one’s attitudes toward risks with positive rewards (Zhang and Palma, 2022), also plays a role in the decision-making of diabetic self-management that involves risks and uncertainties. Utility curvature is associated with better self-care behaviors (Simon-Tuval et al., 2016), better medication adherence (Simon-Tuval et al., 2018; Reach et al., 2018b), and a smaller risk of developing diabetic complications (Emoto et al., 2015). However, previous literature has described an ambiguous association between utility curvature and screening compliance. Some reports have revealed that risk-averse people are more likely to practice risk-reducing health behaviors than are risk-seeking persons (Anderson and Mellor, 2008). However, several studies have found that more risk-averse people are, counterintuitively, less likely to undergo cancer screening, which is a risk-reducing behavior (Picone et al., 2004; Goldzahl, 2017). Thus risk-averse people might be disinclined to receive the potential bad news brought by screening. Other studies have reported null associations. For instance, cancer screening was not associated with a willingness to take risks, neither in general nor in the health domain (Lutter et al., 2019). Given the contradictory evidence, we analyze the effect of utility curvature on screening compliance without assigning any *a priori* sign expectation.

### 2.4. Probability weighting

Probability weighting refers to an individual’s tendency to form subjective probabilities based on objective probabilities (Prelec, 1998; Bernheim and Sprenger, 2020). Empirical evidence shows that the individual-level subjective assessment of risk predicted uptake of flu shots and mammograms (Carman and Kooreman, 2014) and regular cancer screening (Goldzahl, 2017). Picone et al. (2004) developed a theoretical model showing that a higher subjective probability of getting disease increases acceptance of screening. A meta-analysis shows that the prevalence of DR among Chinese persons with diabetes is approximately 23% (Liu et al., 2012), which is a relatively small probability event and is below the reference point or the *status quo* (37%) observed by the experimental studies conducted among Chinese participants (Liu and Huang, 2013). It means that those with diabetes are expected to *over*weight the relatively small probability of contracting DR. A higher probability weighting implies that the probability of contracting DR is distorted to a higher level. Thus, we expect that probability weighting has a positive influence on perceived effectiveness of intervention strategies.

### 2.5. Loss aversion

Loss aversion refers to the notion that people manifest stronger reactions toward losses than objectively commensurate gains (Tversky and Kahneman, 1991; Rouyard et al., 2018; Dogbe and Gil, 2019). Rouyard et al. (2018) inferred loss aversion through its effect on utility under prospect theory and found that loss aversion is an important factor in explaining decision-making in the face of health risks among both people with and without diabetes. Hadlaczky et al. (2018) observed that the more loss-averse individual would place a greater focus on the negative consequences and are less likely to attempt suicide than the less loss-averse counterparts. DR is generally asymptomatic in its early stage but its progression results in irreversible visual impairments, which means that after the emergence of visual impairments patients cannot recover their normal sight even after treatment (Kobrin Klein, 2007). Regular DR screening attendance could also be considered to be self-protective since it can reduce the probability of blindness, a severe health loss. Thus, those with a greater aversion to potential losses are expected to reveal higher screening compliance.

Low participation in DR screening is often explained by income level and education attainment (Emoto et al., 2016; Piyasena et al., 2019), or by lack of knowledge and awareness (Piyasena et al., 2019; Chen et al., 2020), and distance from screening facilities (Lee et al., 2014), etc. In addition, a systematic review has identified behavioral biases that might influence diabetic eye screening (Williams et al., 2018). While greater appreciation of decision-making biases may help us understand low screening rates, and could inform potential interventions, we know little about underlying mechanisms and why some interventions have failed (Tambor et al., 2016; Chen et al., 2018; Judah et al., 2018).

Specifically, in the context of incentive strategies for enhancing uptake of DR screening, the role of behavioral biases has yet to be studied. Thus we aim to explore the influence of risk and time preferences and behavioral biases on decisions about DR screening.

## 3. Experimental design

We utilize a series of monetary time and risk economic experiments to elicit the preference parameters, which have consistently been reported to be predictive of unhealthy behaviors (Story et al., 2014; Dogbe and Gil, 2019). Following Tanaka et al. (2010), we measure risk and time preferences by means of economic experiments with choice lists. Participants are presented with three choice lists that identify their risk preferences and another six choice lists that identify their time preferences (for English translations of the original Chinese documents, see Supplementary Tables S1, S2). The experiments are incentive-compatible, that is, the participants have opportunities to cash out the lotteries after completing the experiments (for lottery boxes see Supplementary Figure 1). Before the commencement of the actual game, the rules were explained to participants in detail, and mock experiments were performed to ensure that they understood the experimental procedures.

### 3.1. Experiments eliciting risk preference parameters

The three choice lists eliciting risk preferences can be found in the Supplementary Table S1. The first two choice lists are designed to estimate participants’ utility curvature and probability weighting. Each row contains two options, offering monetary rewards at different risk levels. Let $\left(x,\;p;\;y,\;1-p\right)$ denote a binary option that gives reward x with probability p and reward y with probability 1−p. Option A is fixed at (¥40, 0.3; ¥10, 0.7) and (¥40, 0.9; ¥30, 0.1) in the first and second task, respectively. Option B offers (x, 0.1; ¥5, 0.9) with reward x varying between ¥70 and ¥600. A rational participant would initially choose the relatively safe option A in the first several rows, but as the reward in option B increases, he/she may switch to option B. To prevent a participant from switching back and forth, monotonic switching is enforced such that participants are asked to indicate the task they would like to switch from option A to option B. Monotonic switching is used to reduce cognitive burden for our older respondents who had rather limited education.

The third task, in which participants encounter the risk of losing money, is used to measure their loss aversion. Both options A and B imply a 50% probability of gaining or losing a certain amount of money, but the gains and losses in option B are relatively larger than that of option A. Progressing from one row to the next, the amount lost increases in option A but gradually decreases in option B, making the latter increasingly attractive. Compared to less loss-averse participants, the more loss-averse would switch from option A to option B at a later stage.

After the completion of the three tasks, each participant draws one ball from a lottery box containing 24 sequentially-numbered balls to decide the number of the choice to be played with real money. Likewise, a similar cash-out game is carried out after the completion of task 3. It should be noted that participants may lose money, but the maximum losses in this game are less than the participation incentive of ¥20 plus the rewards in the other games.

### 3.2. Experiments eliciting time preference parameters

Each participant is presented with six choice lists, with each including 10 choices between a smaller reward paid sooner (option A) and a larger reward delivered after a specific time delay (option B). The designs of these choice lists entail four treatments. Firstly, we apply the front-end delay (FED) treatment, which refers to a month delay to both the early and late payment dates, to avoid placing extra transaction costs on the late payment (Andersen et al., 2008). Secondly, three time-horizons (1 week, 1 month, and 3 months) are used. Thirdly, two reward amounts (¥10 and ¥20) are used to account for the possibility that the time preferences of participants vary across prize sizes. Fourthly, we account for order effects, that is, the order in which the three time-horizons are presented to participants. More specifically, half of our sample are presented with tasks with the smallest to largest time-horizons (i.e., 1 week, 1 month, and 3 months) and the other half with the opposite ordering of time-horizons (i.e., 3 months, 1 month, and 1 week). The four treatments result in 24 choice lists, which are split into four versions, each with 6 choice lists. To reduce the cognitive burden to participants, each is presented with only one version.

Supplementary Table S2 shows two example time tasks. Task 1 (¥10; 1 week) offers intertemporal choices between an immediate reward (¥10) and a later reward (¥11 ~ ¥38) paid in 1 week. In task 2, the payment dates for both options are delayed for 1 week and the reward amount is doubled to ¥20. Within each task, the earlier reward and the time horizon between choices remains identical, but the future reward and the implicit interest rate increase as the participant progresses down to the next choice. Participants have to decide which option is preferred and once again monotonic switching is enforced. The point at which participants switch from option A to option B reveals their potential discount rates. Upon the completion of the tasks, participants are asked to draw one of 60 numbered balls from a lottery box, which determines the number of the choice to be paid. For future payments, participants were given a note stating the date the payment would be paid and contact details of the researchers. For convenience, future payments were paid to participants by topping-up prepaid call credits to the mobile phone number collected during the experiments.

### 3.3. Identification of time and risk preferences

The parameters of risk and time preferences are simultaneously estimated within a Maximum Likelihood (ML) framework by specifying the functional forms of the utility and discount functions. Following Liebenehm and Waibel (2014), we define the utility function under prospect theory as:


(1) $$PT\left(x,\;p;\;y,\;1-p\right)=\pi \left(p\right)v\left(x\right)+\pi \left(1-p\right)v\left(y\right)$$

where $PT\left(x,\;p;\;y,\;1-p\right)$ refers to the expected utilities for the option with $\left(x,\;p;\;y,\;1-p\right)$. The probability weighting function $\pi \left(p\right)$ and value function $v\left(x\right)$ are defined in Equations (2) and (3), respectively:


(2) $$\pi \left(p\right)=1/\exp{\left[\ln\left(1/p\right)\right]}^{\alpha }$$

(3) $$v\left(x\right)=\{\begin{array}{c} x^{\sigma },x\geq 0\\ -\lambda {\left(-x\right)}^{\sigma },x<0 \end{array}$$

where the first risk-preference parameter α determines the shape of the probability weighting function. Note that probability weighting is assumed to be identical for gains and losses because the only probability (i.e., 50%) in Task 3 does not support an empirical estimation of probability weighting of losses. α=1 implies a linear probability weighting function, that is, subjective and objective probabilities are equivalent. Participants tend to overweight small probabilities and underweight large probabilities if α<1, and the departure from linear probability weighting is larger with a smaller α. The second risk-preference parameter σ indicates the concavity of the utility function under risk. A smaller σ implies higher utility curvature, and σ=1 implies risk-neutrality. The third risk-preference parameter of interest is loss aversion (λ) which refers to the notion that people manifest stronger reactions toward losses than objectively commensurate gains. λ>1 implies loss aversion. The above utility functions nest the standard expected utility function, that is, the utility functions collapse to the standard one if α=1 and λ=1.

Regarding the discount function, we utilize the quasi-hyperbolic discount function to capture the conventional discount rate and time-inconsistent discounting. The quasi-hyperbolic discount function $D\left(t\right)$ for immediate $\left(t=0\right)$ and delay $\left(t>0\right)$ rewards is defined as the following:


(4) $$D\left(t\right)=\left\{\begin{array}{c} 1,\;t=0\\ \beta e^{-\delta t},\;t>0 \end{array}\right.$$

where δ denotes the conventional discount rate and β captures present-bias. Equation (4) reduces to exponential discount function if β=1 (i.e., no present-bias). A higher δ and β imply a higher discount rate and a smaller present-bias, respectively. After integrating the utility and discount functions into a structural framework, the utility ($U_{i}^{j}$) for participant i gained from the choice task j in the risk and time experiments are defined as follows:


(5) $$U_{i}^{j}=D_{i}^{j}\left(\tau +t\right)PT_{i}^{j}\left(x,p\right)+\varepsilon_{ij}$$

where $D_{i}^{j}\left(.\right)$ and $PT_{i}^{j}\left(.\right)$ refer to the discount and utility functions, respectively. t represents the difference (in days) of payment delay in options A and B. τ=30 refers to the one-month front-end delay and τ=0 implies no such delay. x and p represent reward amounts and probabilities, respectively. $\varepsilon_{ij}$ is an *i.i.d.* error term. It should be noted that Equation (5) nests both time and risk experiments. For instance, the utilities for the choice task in the time experiment can be obtained by assuming payments with certainty (i.e., p=0). Similarly, the utilities for the options in the risk experiments can be calculated by assuming immediate payments (i.e., τ=t=0).

The log-likelihood function can be constructed using either all binary choices (Tanaka et al., 2010) or a certainty equivalent calculated as the expectations of the rewards of the two options adjacent to the switching point of each choice list (Bruhin et al., 2010). Given the relatively small number of choice lists, we opt for the former approach to maximize the number of observations and to fully utilize the reward values across all binary choices. Participants are expected to opt for option A and not option B if the former generates a higher utility, and vice versa. The difference between the utility streams under the two options can be defined as $I_{i}^{j}=U_{i}^{B;j}-U_{i}^{A;j}$, where $U_{i}^{B;j}$ and $U_{i}^{A;j}$ refer to the utility of option B and A for the participant i with the choice task j, respectively. The conditional log-likelihood function for participant i can be expressed as:


(6) $$\ln L_{i}\left(\alpha ;\sigma ;\lambda ;\delta ;\beta ;y_{i};X_{i}\right)=\overset{91}{\underset{j=1}{\sum }}\left\{\begin{array}{l} \left[ln\phi \left(-I_{i}^{j}\right)|y_{i}^{j}=0\right]+\\ \left[ln\phi \left(I_{i}^{j}\right)|y_{i}^{j}=1\right] \end{array}\right\}$$

where α, σ, λ, δ, and β are the five risk and time preference parameters defined above. The number 91 refers to the total number of choices across all tasks. $y_{i}^{j}=0$ and $y_{i}^{j}=1$ refer to the choice of option A and B by participant i in the jth task, respectively. Equation (6) can be maximized using standard numerical methods to obtain the five preference parameters. Sub-group analyses are conducted separately for the urban and rural samples, and cross-group differences in parameters are tested using t-statistics. Next, in the heterogeneity analysis, each preference parameter is assumed to be a linear function of the participants’ perceived effectiveness of incentive strategies and their socio-demographic characteristics within the same maximum likelihood framework. All statistical analyses were carried out using Stata, version 16.0 (Stata Corp., College Station, Texas).

## 4. Data collection and descriptive statistics

### 4.1. Sample selection

In July 2015, a field study was conducted in rural and urban areas of Guangdong, southern China. Urban dwellers with diabetes were randomly selected from a pool of older patients aged 50+ registered in the database of the Longfeng Community Healthcare Center, Haizhu district, Guangzhou city. Rural dwellers were sampled from a group of 517 older patients living with diabetes identified in the population-based Yangxi Eye Study conducted in rural Yangxi county. A two-stage randomly-stratified sampling procedure was employed. At the first stage, a total of 24 villages were ranked according to their proximity to the central hospital of Yangxi. Six villages from the stratum at greatest distance and another six villages from the nearest stratum were selected. At the second stage, we randomly selected 4–5 gender-stratified participants within a village from a list of previously-identified older patients. A pre-survey pilot was conducted among a group of four participants to improve the flow of economic experiments and to identify any inconsistencies in the questionnaire. Each subject gave written informed consent and was offered a participation incentive of ¥40. This study was approved by the ethics committee of Zhongshan Ophthalmic Center (approval number: 2015MEKY069). A total of 177 persons living with diabetes participated in the survey. Eleven participants failed to understand the economic experiments after repeated explanation, and were excluded from the analysis, resulting in a final analytic sample of 166.

### 4.2. Sample characteristics

Table 1 presents the characteristics and descriptive statistics of the sample. The mean age is 61.26 years and does not vary across urban and rural areas. Gender is almost evenly distributed with 48% males and 52% females. 60% of our sample live in rural areas and the remaining 40% in urban areas. The participants have generally low levels of education (only just over primary school education). They also reportedly have a low income between 501 and 1,000 yuan and 1,001–2000 yuan. The average travel time to the nearest hospital is approximately half an hour. The mean family size is 4.19 and the known diabetes duration is 4.71 years on average.

**Table 1:** Descriptive statistics of variables.

Variables	Definitions	*N*	Mean	S.D.	Min.	Max.
Perceived effectiveness	Sum of the scores of each perceived effectiveness item	166	41.24	9.43	11	55
Rural	= 1 if participant is in the rural sample, and 0 in the urban sample	166	0.60	0.49	0	1
Age	Age of participants (in years)	166	61.26	6.07	50	73
Male gender	= 1 if participant is male, and 0 otherwise	166	0.48	0.50	0	1
Education	= 1 if “No education”; =2 if “Less than primary school”; =3 if “Finished primary school”; =4 if “Secondary school”; =5 if “High school or higher.”	166	3.18	1.43	1	5
Income	= 1 if income less than 500; =2 if 501–1,000; =3 if 1,001–2000; =4 if 2001–3,000; =5 if above 3,000.	166	2.69	1.60	1	5
Family size	= 1 if “live alone”; =2 if “live with 1 person”; =3 if “live with 2 people”; =4 if “live with 3 people”; =5 if “live with 4 people”; =6 if “live with 5–6 people”; =7 if “live with 7–8 people”; and = 8 if “live with 9 or more people.”	166	4.19	1.95	1	8
Travel time to nearest hospital	Travel time to the nearest hospital from home (in minutes)	166	30.81	23.81	2	180
Known diabetes duration	Duration after being diagnosed with diabetes (in years)	166	4.71	6.07	1	30

### 4.3. Measurement of perceived effectiveness of incentive strategies

Participants’ perceptions of the effectiveness of incentive strategies were measured with an 11-item questionnaire which asked them to respond to a series of questions “*How effective do you think each of the following incentive strategies is in promoting your adherence to an annual eye examination?*” Each item was scored on a 5-point Likert scale ranging from 1 “very ineffective” to 5 “very effective.” The 11 incentive strategies include the provision of: (1) having a nurse/doctor contact you every 6 months; (2) receiving an automatic text reminder on your cellphone; (3) watching a video and getting other information about diabetes and the eye; (4) seeing a picture of the back of your eye at each examination; (5) free transportation to the hospital; (6) cash payment; (7) free medicine; (8) cellphone top-ups; (9) free textbooks, eye exam, or glasses for any child in the extended family; (10) watching videos and posters that show new, high-quality equipment used for eye checking and the training certificates of doctors caring for you; (11) free eye check. We obtained a perceived effectiveness scale equal to the sum of each item score. Perceived effectiveness has a mean of 44.97, and urban samples (46.30) have a significantly higher perceived effectiveness than their rural counterparts (44.09).

## 5. Results

### 5.1. Perceived effectiveness of incentive strategies

Table 2 presents data on the perceived effectiveness of incentive strategies. Overall, participants perceived a free eye check as the most effective incentive strategy (mean 4.37) and receiving an automatic text reminder as the least effective (mean 3.36). Urban and rural participants have different attitudes toward incentive strategies. The mean ratings for the perceived effectiveness of incentive strategies by urban participants (3.92) are significantly higher (*p* < 0.05) than that (3.64) of rural participants, implying that urban participants are more receptive toward incentives than their rural counterparts.

**Table 2:** Descriptive statistics of the perceived effectiveness of various incentive strategies.

Items		Pooled		Urban		Rural		Differences
		Mean	Std. Dev.	Mean	Std. Dev.	Mean	Std. Dev.	
1	Having a nurse/doctor contact you every 6 months.	4.06	1.26	4.47	0.83	3.79	1.42	0.68***
2	Receiving an automatic text reminder on your cellphone before your exam.	3.36	1.58	4.29	1.11	2.74	1.54	1.55***
3	Watching a video and getting other information about diabetes and the eye.	3.37	1.38	4.17	0.94	2.84	1.37	1.33***
4	Seeing a picture of the back of your eye at each inspection, so you could tell if you were getting better or worse.	3.89	1.24	4.62	0.70	3.41	1.28	1.21***
5	Getting free transportation to the hospital.	3.73	1.33	3.20	1.42	4.08	1.15	−0.88***
6	Being paid in cash every time you came back to the hospital for care.	3.89	1.28	3.47	1.45	4.16	1.07	−0.69***
7	Giving free medicine every time you came back to the hospital for care.	3.94	1.32	3.62	1.49	4.15	1.16	−0.53**
8	Receiving some call credits.	3.79	1.32	3.59	1.37	3.92	1.28	−0.33
9	Providing a free school year of textbooks, a free eye exam, and free glasses as needed for any child in the extended family.	3.46	1.39	3.41	1.47	3.50	1.34	−0.09
10	Watching videos and posters that show new, high-quality equipment used for eye checking and the training certificates of doctors caring for DR.	3.39	1.28	3.82	1.28	3.11	1.21	0.71***
11	Free eye check.	4.37	1.05	4.45	1.03	4.31	1.06	0.14
Perceived effectiveness		44.97	10.38	46.30	9.57	44.09	10.83	3.10**

***p < 0.01, **p < 0.05, and *p < 0.1.

### 5.2. Estimated time and risk preference parameters

Table 3 presents the estimated time and risk preference parameters. Regarding the time preference parameters, the estimated discount rate (δ) is 0.882. The mean estimated β (0.800) is significantly smaller than 1, implying strong present bias. The estimate of the utility curvature parameter (σ =0.284) indicates that participants are generally risk-averse. Also, it can be concluded that participants are in general loss-averse, because the estimated λ (2.354) is significantly larger than 1. The parameter for probability weighting (α) has a mean of 0.304, suggesting that participants tend to overweight small probabilities and underweight large probabilities. These results demonstrate the appropriateness of the utility curvature and quasi-hyperbolic discount functional specifications.

**Table 3:** Estimates of risk and time parameters.

	Pooled sample	Urban	Rural
Time preference (δ)	0.882***	0.955***	0.829***
	(0.17)	(0.20)	(0.27)
Present bias (β)	0.800***	0.820***	0.776***
	(0.03)	(0.03)	(0.04)
Utility curvature (σ)	0.284***	0.331***	0.251***
	(0.01)	(0.02)	(0.02)
Probability weighting (α)	0.304***	0.340***	0.263***
	(0.05)	(0.05)	(0.08)
Loss aversion (λ)	2.354***	1.637***	3.097***
	(0.16)	(0.18)	(0.29)
Observations	15,106	6,006	9,100
Tests	*p*-value		
H_0_: α =1	0.000	0.000	0.000
H_0_: λ =1	0.000	0.000	0.000
H_0_: β =1	0.000	0.000	0.000

Parentheses in parentheses are standard errors clustered at the individual level. *** *p* < 0.01, ** *p* < 0.05, and * *p* < 0.1.

The estimated parameters differ between urban and rural areas. Urban participants (0.955) have significantly (*p* < 0.01) higher discount rates than rural residents (0.829). Urban participants (β =0.820) are subject to significantly (*p* < 0.001) less present-bias compared to rural participants (β =0.776). Further, compared to their urban counterparts (σ =0.331; λ =1.637), rural participants (σ =0.251; λ =3.097) are more risk-averse (p < 0.001) and loss-averse (p < 0.001). Finally, rural participants (0.263) have a significantly (*p* < 0.001) higher probability weighting than the urban participants (0.340), suggesting that the subjective probabilities of the former group differ from objective probabilities to a larger extent than the latter (Table 3).

### 5.3. Association between risk and time preferences and perceived effectiveness of incentive strategies

Discount rates are negatively (−0.050, *p* < 0.05) associated with *Perceived effectiveness*, implying that those living with diabetes who place a lower value on future benefits perceive incentive strategies to have smaller effects than those who are more patient (Table 4). The coefficient of present-bias (0.002, *p* > 0.10) has a positive but insignificant sign, which does not support our hypothesis that those with less self-control problems have higher perceived effectiveness of incentive strategies. Next, risk preference has an insignificant coefficient of 0.002 (*p* > 0.10), implying that willingness to take risks is not associated with perceived effectiveness of incentive strategies. Loss aversion is negatively and significantly (−0.043, *p* < 0.05) related to *Perceived effectiveness*, suggesting that participants with higher levels of loss aversion are more difficult to motivate with incentives. Finally, the composite measure of perceived effectiveness of incentive strategies is positively and significantly (0.019, *p* < 0.05) associated with the probability weighting parameter α, which indicates that the participants with a higher level of probability weighting are less convinced of the usefulness of incentive strategies, compared to participants with a lower probability weighting (Table 4).

**Table 4:** Association between risk and time preferences and perceived effectiveness of intervention strategies.

	Discount rate (δ)	Present- bias (β)	Utility curvature (σ)	Probability weighting (α)	Loss aversion (λ)
Perceived effectiveness	−0.050**	0.002	0.002	0.019**	−0.043*
	(0.02)	(0.00)	(0.00)	(0.01)	(0.02)
Rural	−0.545	−0.081	0.075	1.280***	1.429
	(0.51)	(0.12)	(0.05)	(0.20)	(0.94)
Age	−0.024	−0.000	−0.000	0.006	−0.026
	(0.03)	(0.01)	(0.00)	(0.02)	(0.04)
Gender	0.008	−0.035	−0.064**	−0.185	0.361
	(0.32)	(0.06)	(0.03)	(0.12)	(0.48)
Education	−0.033	−0.045	0.049**	0.431***	0.469
	(0.16)	(0.04)	(0.02)	(0.07)	(0.36)
Income	−0.172	0.030	0.004	0.277***	−0.104
	(0.14)	(0.04)	(0.02)	(0.05)	(0.21)
Family size	0.024	−0.006	0.003	0.037	−0.048
	(0.09)	(0.02)	(0.01)	(0.03)	(0.13)
Travel time	0.004	0.004	−0.001	−0.001	0.003
	(0.01)	(0.00)	(0.00)	(0.00)	(0.01)
Known diabetes duration	0.013	0.000	0.001	0.010	−0.081
	(0.02)	(0.01)	(0.00)	(0.01)	(0.05)
Constant	5.274**	0.728	0.066	−3.096**	4.213
	(2.42)	(0.46)	(0.21)	(1.25)	(3.54)
Number of clusters	166				
Number of observations	15,106				
Log pseudo-likelihood	−9293.52				

Parentheses are standard errors clustered at the individual level. *** *p* < 0.01, ** *p* < 0.05, and * *p* < 0.1.

### 5.4. Control variables

Table 4 also reports the associations between the socio-demographic variables and preference parameters. Those who lived in rural areas and had higher education and income had significantly larger probability weighting parameters. Males were less risk-averse than females, a finding consistent with Charness and Gneezy (2012). Those individuals with higher education are more risk-averse than the less educated, which is in line with Liebenehm and Waibel (2014). Finally, none of the socio-demographic variables were significantly associated with loss aversion, discount rate, and present-bias.

### 5.5. Sub-group analysis

We observe some heterogeneity regarding the associations between perceived effectiveness of incentive strategies and some, but not all, preference parameters (Table 5). The relationship between *Perceived effectiveness* and discount rate (δ) is present for rural (−0.058, *p* < 0.05) but not for rural participants (−0.015, *p* > 0.10). Likewise, present-bias is significantly associated with *Perceived effectiveness* among the urban (0.008, *p* < 0.10) but not among the rural (0.000, *p* > 0.10) participants. In contrast, for both rural (−0.080, *p* < 0.10) and urban (−0.059, *p* < 0.05) participants, those with higher levels of loss aversion are more difficult to motivate with incentives. Similarly, the lack of association between risk preference and *Perceived effectiveness* in the pooled sample analysis also applies to the two sub-groups. Notably, the probability weighting parameter is positively associated with *Perceived effectiveness* among rural participants (0.015, *p* < 0.05), in contrast to the opposite association among urban residents (−0.038, *p* < 0.01).

**Table 5:** Association between risk and time preferences and perceived effectiveness of intervention strategies.

Variables	Rural					Urban				
	Discount rate (δ)	Present bias (β)	Utility curvature (σ)	Probability weighting (α)	Loss aversion (λ)	Discount rate (δ)	Present bias (β)	Utility curvature (σ)	Probability weighting (α)	Loss aversion (λ)
Perceived effectiveness	−0.058**	0.000	0.002	0.015**	−0.080*	−0.015	0.008*	0.002	−0.038***	−0.059**
	(0.03)	(0.00)	(0.00)	(0.01)	(0.05)	(0.03)	(0.00)	(0.00)	(0.01)	(0.03)
Age	−0.031	−0.004	−0.001	0.007	0.006	0.017	0.005	−0.000	−0.013	−0.037
	(0.04)	(0.01)	(0.00)	(0.02)	(0.05)	(0.06)	(0.01)	(0.01)	(0.02)	(0.04)
Gender	0.409	0.009	−0.069	−0.127	0.738	−0.610	−0.109	−0.123*	−0.456***	−0.078
	(0.58)	(0.09)	(0.04)	(0.12)	(0.69)	(0.57)	(0.08)	(0.07)	(0.12)	(0.42)
Education	−0.043	−0.025	0.030	0.507***	0.888	−0.396	−0.051	0.101**	−0.166	−0.086
	(0.23)	(0.05)	(0.03)	(0.13)	(0.66)	(0.46)	(0.08)	(0.05)	(0.13)	(0.49)
Income	−0.129	0.029	0.015	0.287**	0.472	−0.276	0.007	0.120	0.015	−0.107
	(0.21)	(0.04)	(0.02)	(0.13)	(0.36)	(0.42)	(0.07)	(0.08)	(0.18)	(0.45)
Family size	0.023	−0.008	0.005	0.041	−0.144	−0.249	−0.065	0.023	−0.046	0.009
	(0.13)	(0.02)	(0.01)	(0.03)	(0.17)	(0.18)	(0.05)	(0.02)	(0.08)	(0.17)
Travel time	0.008	0.011***	−0.001	0.002	0.014	0.002	−0.001	0.002*	0.009	−0.006
	(0.02)	(0.00)	(0.00)	(0.00)	(0.03)	(0.00)	(0.00)	(0.00)	(0.01)	(0.01)
Known diabetes duration	−0.180	−0.035***	0.020***	0.094***	−0.254	0.028	0.015	−0.005	−0.055***	0.024
	(0.14)	(0.01)	(0.01)	(0.01)	(0.20)	(0.03)	(0.01)	(0.00)	(0.02)	(0.03)
Constant	5.425	0.742	0.211	−2.030*	4.009	4.587	0.600	−0.695	4.593**	7.310
	(3.39)	(0.49)	(0.28)	(1.13)	(3.45)	(5.74)	(0.85)	(0.52)	(1.94)	(5.30)
Number of clusters	100					66				
Number of observations	9,100					6,006				
Log pseudo-likelihood	−5451.70					−3452.44				

Parentheses are standard errors clustered at the individual level. *** *p* < 0.01, ** *p* < 0.05, and * *p* < 0.1.

## 6. Discussion and implications

Our observed discount rate of 0.88 is identical to that found by Wang and Sloan (2018) in a study on US residents, but differs from several other studies on rural dwellers (0.37) from West Africa (Liebenehm and Waibel, 2014) and Vietnam (2.92) (Tanaka et al., 2010). We find that a higher discount rate is significantly and systematically associated with weaker perceived effectiveness of incentive strategies, which differs from a recent study by Lipman (2020) reporting a null association between discount rate and students’ preferences for receiving financial incentives for exercise. The following considerations may explain the discrepancy. First, behavioral economic theories assume that people with higher discount rates place a lower value on future benefits, and thus are more difficult to motivate with incentives for a behavioral change with little immediate utility. Second, we have based our analysis on a group of people with chronic disease, (as opposed to a cohort of students), who face real-life decisions about whether to adhere to screening. Third, we have used real incentives in contrast to Lipman (2020) using hypothetical incentives.

Our finding of a present-biased sample is in line with most studies based on experimental methods, although the extent of present-bias varies from study to study. For instance, β is variously estimated as 0.51–0.79 (Fang and Wang, 2015), 0.94 (Liebenehm and Waibel, 2014), 0.64 (Tanaka et al., 2010), and 0.38 (Wang and Sloan, 2018). However, it is surprising to find that present-bias is not associated with the perceived effectiveness of financial incentives in the pooled sample analysis. A possible explanation is that self-evaluation of incentive strategies does not predict the self-control problem (i.e., present-bias) which can only be manifest when faced with an immediate decision. Those with self-control problems might procrastinate about screening to avoid immediate efforts, despite their preferences for future benefits (Matjasko et al., 2016), but this preference reversal cannot be tested with the current protocol, based on hypothetical, rather than real, incentives.

Our finding of apparent loss aversion in this cohort (estimated parameter 2.35) is consistent with other studies, including Liebenehm and Waibel’s estimate of 1.35 (2014) and Tanaka *et al*’s value of 2.63 (2010). The fact that more loss-averse participants have lower perceived effectiveness of incentive strategies than less loss-averse ones appears counter-intuitive. One possible explanation is that people with diabetes might perceive cost and time spent on screening as losses if no retinopathy is identified, despite the fact that regular prophylactic examination might facilitate early detection and timely treatments. This might also be a result of the psychological burden attendant on older patients discussing illnesses with family members who will assume the burden of caring for them (Duan et al., 2017). Next, DR screening attendance has the potential to deliver bad news, which could provoke anxiety or stress, and come at a psychological cost to the individual. Those who are more loss-averse might be more willing to avoid the potential bad news triggered by screening.

Next, the finding that loss aversion, but not utility curvature, is related to perceived effectiveness of incentive strategies also deserves attention. Our finding is inconsistent with Goldzahl (2017) who showed that utility curvature increased the likelihood of regular breast cancer screening. Nevertheless, this might be explained by the conjecture that some health decisions relate more to health losses than to gains, but it is more generally accepted in the medical literature that loss framing is more effective (than gain framing) for screening decisions while positive framing (emphasizing chances of survival) is more effective than negative framing (regarding chances of death) for influencing choices among risky options (Edwards et al., 2002). Of course other psychological processes may be at play, such as regret minimization, which, in this instance, we have not explicitly investigated (Boeri et al., 2013). In line with prospect theory, participants in our study *over*-weight small probabilities and *under*-weight large ones, meaning that the relatively small objective probabilities of experiencing DR are likely to be overweighed. Our estimate of the probability weighting parameter (0.304) is larger than that (0.11) reported by Liebenehm and Waibel (2014) and smaller than that (0.59) in Tanaka et al. (2010). The overweighting of the possibility of disease is consistent with Goldzahl (2017) who found that a majority of women respondents tend to overestimate their risks of developing breast cancer. Next, the pooled sample analysis showed that a higher subjective weighting of probabilities (i.e., smaller α) is significantly and negatively associated with perceived effectiveness of incentive strategies. This finding runs counter to our hypothesis. However, this seemingly counterintuitive finding only applies to the rural residents. A potential explanation is that, compared to urban residents, rural residents have much higher probabilities of developing DR (Liu et al., 2012). The probabilities are likely to exceed the reference point, above which probabilities go from over-weighted to under-weighted. For the rural sample, the increase of the preference parameter α implies that relatively large probabilities will be *under*weighted to a smaller extent, which in turn indicates higher perceived effectiveness of incentive strategies. In contrast, the sub-group analyses on urban residents showed that a higher subjective weighting of probabilities (i.e., smaller α) increases with perceived effectiveness of incentive strategies, which is in line with our expectation. The reason is that the probability of developing DR among the urban group is below the reference point. Our finding of group heterogeneity is consistent with the review by Rouyard et al. (2017) who have observed mixed results regarding the perception of risk of contracting diabetes-related complications among people with diabetes, with some studies reporting an overestimation of risk while others under-estimated it.

Our finding that people with diabetes who have higher discount rates, who are more loss averse, and who have lower decision weights perceive incentive strategies to be less effective has implications for the tailoring of incentive based interventions for encouraging DR screening. Identifying specific behavioral biases and targeting the identified biases should provide more effective interventions to increase DR screening. For instance, existing studies have established the associations between higher discount rates and the onset of diabetes, poorer diabetes self-management, and higher glycemic levels. However, in a randomized trial, incentives were ineffective in promoting diabetic eye screening (Judah et al., 2018). In a broader context, the findings around the effectiveness of incentives in promoting health-related behavior among older people are inconsistent. This might be because universal incentive levels would not take account of the heterogeneity in individual-specific time preferences. Previous studies have demonstrated the effectiveness of loss aversion-framed incentives in motivating weight loss (Volpp et al., 2008) and smoking cessation (Halpern et al., 2015), however, these incentives were not tailored according to individual discount rates. Based on insights from our study, we argue that incentives should be tailored to the discount rates of patients living with diabetes. Further, although mobile phone reminders are effective in improving adherence to DR screening among patients with diabetes compared to control groups without reminders (Chen et al., 2018), over half of the patients in the intervention group were still absent from their scheduled appointments. We conjecture that the incentive strategies might have negative consequences, that is, the enhanced biases toward the perceived risks from screening. Health communication strategies should emphasize the low risk of screening itself. Tailoring messages to one’s loss aversion might be needed to design more effective public health interventions that further increase the compliance rate.

We find a strong urban vs. rural heterogeneity in the association between our behavioral economic concepts and the perceived effectiveness of intervention strategies. For instance, a higher discount rate is associated with lower perceived effectiveness among rural but not urban participants, while in contrast, a higher present-bias is related to lower perceived effectiveness among urban but not rural participants. These findings point to the need for differentiated intervention strategies tailored to urban and rural patients with diabetes.

The finding that some but not all behavioral biases are associated with perceived effectiveness of incentive strategies highlights the considerable complexity involved in their mechanisms of action. Designing behavior change techniques based on the understanding of the potential processes through which a behavior change technique affects behavior is crucial for the usefulness of these intervention techniques (Michie et al., 2021). Although low compliance with preventive care is an international phenomenon, there are still apparent discrepant findings regarding the impact and mechanisms of incentive based interventions which are likely to be context and population dependent. Thus, future studies should aim to further understand how incentive-based behavioral change interventions trigger composite mechanisms targeting multiple behavioral biases. This could generate a deeper understanding of the context-mechanism-outcome configurations that might improve intervention implementation (Vanderkruik and McPherson, 2017).

Although we have established the relationship between time preference and perceived effectiveness of potential incentive strategies, one limitation of the present study is that hypothetical survey questions were used to measure perceived effectiveness. We were unable to test for the effectiveness of a real-life incentive scheme that tailors incentives to the behavioral economic traits of people with diabetes. We have demonstrated that the relationship between behavioral economic traits, risk preference and incentives to enhance screening is complex and so the design of integrated interventions will require careful intervention mapping (Wight et al., 2016; Lake et al., 2018) and tailoring to the individual and context, especially when there exists the possibility that changes in executive function (from poor diabetic control) might impact the very behavioral mechanisms that you are trying to influence (Epstein et al., 2019). There is clearly scope and opportunity for such novel interventions, as a recent HTA review of conventional psychological support interventions found minimal benefit for diabetic self-management (Winkley et al., 2020). There is already an established literature supporting interventions informed by behavioral economic traits, such as Episodic Future Thinking (Epstein et al., 2022) and framing and commitment devices (Kullgren et al., 2017; Göllner et al., 2018) but public health practitioners trying to improve the outcomes for people with chronic disease must also recognize that upstream determinants such as poverty and material disadvantage also affect behavioral economic traits and decision making agency. Thus “personalized prevention” must be balanced with whole population approaches to mitigate increasing inequalities (Taylor-Robinson and Kee, 2018). Randomized controlled trials would be required to fully demonstrate the usefulness of these approaches. In addition, we focused on the roles of the types of incentives, but we did not study the association between economic preferences and different dimensions of incentives, such as framing, timing, increasing payoffs, etc. This could be an important direction for future research.

## Data availability statement

The raw data supporting the conclusions of this article will be made available by the authors, without undue reservation.

## Ethics statement

The studies involving human participants were reviewed and approved by Ethics committee of Zhongshan Ophthalmic Center (approval number: 2015MEKY069). The patients/participants provided their written informed consent to participate in this study.

## Author contributions

JT designed the study, collected data, and drafted the manuscript. ZY analyzed the data. FK and NC obtained funding for the study and supervised data collection. All authors critically revised the manuscript for intellectual content and approved the final manuscript. JT is the guarantor of this work and, as such, had full access to all the data in the study and takes responsibility for the integrity of the data and the accuracy of the data analysis. All authors contributed to the article and approved the submitted version.

## Funding

This study is supported by Public Health & Disease Control and Prevention, Major Innovation & Planning Interdisciplinary Platform for the “Double-First Class” Initiative, Renmin University of China.

## Conflict of interest

The authors declare that the research was conducted in the absence of any commercial or financial relationships that could be construed as a potential conflict of interest.

## Publisher’s note

All claims expressed in this article are solely those of the authors and do not necessarily represent those of their affiliated organizations, or those of the publisher, the editors and the reviewers. Any product that may be evaluated in this article, or claim that may be made by its manufacturer, is not guaranteed or endorsed by the publisher.
