# Supplementary material for: Time and risk preferences and the perceived effectiveness of incentives to comply with diabetic retinopathy screening among older adults with type 2 diabetes
Source: Front Psychol. 2023 Apr 17;14:1101909. doi: 10.3389/fpsyg.2023.1101909 (PMC10149913; doi:10.3389/fpsyg.2023.1101909)
Supplement: Supplementary file 1 [file Data_Sheet_1.docx]

Supplementary Material

# ****Supplementary Table S1****. Tasks to elicit risk preferences

| Choice | Option A | Option B | Circle your choice |
| --- | --- | --- | --- |
| Task 1 |  |  |  |
| 1 | Receive ¥40 if drawn ball 1-3; Receive ¥10 if drawn ball 4-10. | Receive ¥70 if drawn ball 1; Receive ¥5 if drawn ball 2-10. | A or B |
| 2 | Receive ¥40 if drawn ball 1-3; Receive ¥10 if drawn ball 4-10. | Receive ¥75 if drawn ball 1; Receive ¥5 if drawn ball 2-10. | A or B |
| 3 | Receive ¥40 if drawn ball 1-3; Receive ¥10 if drawn ball 4-10. | Receive ¥85 if drawn ball 1; Receive ¥5 if drawn ball 2-10. | A or B |
| 4 | Receive ¥40 if drawn ball 1-3; Receive ¥10 if drawn ball 4-10. | Receive ¥95 if drawn ball 1; Receive ¥5 if drawn ball 2-10. | A or B |
| 5 | Receive ¥40 if drawn ball 1-3; Receive ¥10 if drawn ball 4-10. | Receive ¥105 if drawn ball 1; Receive ¥5 if drawn ball 2-10. | A or B |
| 6 | Receive ¥40 if drawn ball 1-3; Receive ¥10 if drawn ball 4-10. | Receive ¥125 if drawn ball 1; Receive ¥5 if drawn ball 2-10. | A or B |
| 7 | Receive ¥40 if drawn ball 1-3; Receive ¥10 if drawn ball 4-10. | Receive ¥150 if drawn ball 1; Receive ¥5 if drawn ball 2-10. | A or B |
| 8 | Receive ¥40 if drawn ball 1-3; Receive ¥10 if drawn ball 4-10. | Receive ¥180 if drawn ball 1; Receive ¥5 if drawn ball 2-10. | A or B |
| 9 | Receive ¥40 if drawn ball 1-3; Receive ¥10 if drawn ball 4-10. | Receive ¥220 if drawn ball 1; Receive ¥5 if drawn ball 2-10. | A or B |
| 10 | Receive ¥40 if drawn ball 1-3; Receive ¥10 if drawn ball 4-10. | Receive ¥300 if drawn ball 1; Receive ¥5 if drawn ball 2-10. | A or B |
| 11 | Receive ¥40 if drawn ball 1-3; Receive ¥10 if drawn ball 4-10. | Receive ¥400 if drawn ball 1; Receive ¥5 if drawn ball 2-10. | A or B |
| 12 | Receive ¥40 if drawn ball 1-3; Receive ¥10 if drawn ball 4-10. | Receive ¥600 if drawn ball 1; Receive ¥5 if drawn ball 2-10. | A or B |
| Task 2 |  |  |  |
| 13 | Receive ¥40 if drawn ball 1-9; Receive ¥30 if drawn ball 10. | Receive ¥70 if drawn ball 1; Receive ¥5 if drawn ball 2-10. | A or B |
| 14 | Receive ¥40 if drawn ball 1-9; Receive ¥30 if drawn ball 10. | Receive ¥75 if drawn ball 1; Receive ¥5 if drawn ball 2-10. | A or B |
| 15 | Receive ¥40 if drawn ball 1-9; Receive ¥30 if drawn ball 10. | Receive ¥85 if drawn ball 1; Receive ¥5 if drawn ball 2-10. | A or B |
| 16 | Receive ¥40 if drawn ball 1-9; Receive ¥30 if drawn ball 10. | Receive ¥95 if drawn ball 1; Receive ¥5 if drawn ball 2-10. | A or B |
| 17 | Receive ¥40 if drawn ball 1-9; Receive ¥30 if drawn ball 10. | Receive ¥105 if drawn ball 1; Receive ¥5 if drawn ball 2-10. | A or B |
| 18 | Receive ¥40 if drawn ball 1-9; Receive ¥30 if drawn ball 10. | Receive ¥125 if drawn ball 1; Receive ¥5 if drawn ball 2-10. | A or B |
| 19 | Receive ¥40 if drawn ball 1-9; Receive ¥30 if drawn ball 10. | Receive ¥150 if drawn ball 1; Receive ¥5 if drawn ball 2-10. | A or B |
| 20 | Receive ¥40 if drawn ball 1-9; Receive ¥30 if drawn ball 10. | Receive ¥180 if drawn ball 1; Receive ¥5 if drawn ball 2-10. | A or B |
| 21 | Receive ¥40 if drawn ball 1-9; Receive ¥30 if drawn ball 10. | Receive ¥220 if drawn ball 1; Receive ¥5 if drawn ball 2-10. | A or B |
| 22 | Receive ¥40 if drawn ball 1-9; Receive ¥30 if drawn ball 10. | Receive ¥300 if drawn ball 1; Receive ¥5 if drawn ball 2-10. | A or B |
| 23 | Receive ¥40 if drawn ball 1-9; Receive ¥30 if drawn ball 10. | Receive ¥400 if drawn ball 1; Receive ¥5 if drawn ball 2-10. | A or B |
| 24 | Receive ¥40 if drawn ball 1-9; Receive ¥30 if drawn ball 10. | Receive ¥600 if drawn ball 1; Receive ¥5 if drawn ball 2-10. | A or B |
| Task 3 |  |  |  |
| 1 | Receive ¥25 if drawn ball 1-5; Lose ¥4 if drawn ball 6-10. | Receive ¥30 if drawn ball 1-5; Lose ¥21 if drawn ball 6-10. | A or B |
| 2 | Receive ¥4 if drawn ball 1-5; Lose ¥4 if drawn ball 6-10. | Receive ¥30 if drawn ball 1-5; Lose ¥21 if drawn ball 6-10. | A or B |
| 3 | Receive ¥1 if drawn ball 1-5; Lose ¥4 if drawn ball 6-10. | Receive ¥30 if drawn ball 1-5; Lose ¥21 if drawn ball 6-10. | A or B |
| 4 | Receive ¥1 if drawn ball 1-5; Lose ¥4 if drawn ball 6-10. | Receive ¥30 if drawn ball 1-5; Lose ¥16 if drawn ball 6-10. | A or B |
| 5 | Receive ¥1 if drawn ball 1-5; Lose ¥8 if drawn ball 6-10. | Receive ¥30 if drawn ball 1-5; Lose ¥16 if drawn ball 6-10. | A or B |
| 6 | Receive ¥1 if drawn ball 1-5; Lose ¥8 if drawn ball 6-10. | Receive ¥30 if drawn ball 1-5; Lose ¥14 if drawn ball 6-10. | A or B |
| 7 | Receive ¥1 if drawn ball 1-5; Lose ¥8 if drawn ball 6-10. | Receive ¥30 if drawn ball 1-5; Lose ¥11 if drawn ball 6-10. | A or B |

# ****Supplementary Table S2****. Tasks to elicit time preferences

| Choice | Option A | Option B | Circle your choice | |
| --- | --- | --- | --- | --- |
| Task 1 (no FED) | | | |  |
| 1 | ¥10 | ¥11 in 1 week | A or B | |
| 2 | ¥10 | ¥14 in 1 week | A or B | |
| 3 | ¥10 | ¥17 in 1 week | A or B | |
| 4 | ¥10 | ¥20 in 1 week | A or B | |
| 5 | ¥10 | ¥23 in 1 week | A or B | |
| 6 | ¥10 | ¥26 in 1 week | A or B | |
| 7 | ¥10 | ¥29 in 1 week | A or B | |
| 8 | ¥10 | ¥32 in 1 week | A or B | |
| 9 | ¥10 | ¥35 in 1 week | A or B | |
| 10 | ¥10 | ¥38 in 1 week | A or B | |
| Task 2 (with one-month FED) | | | |  |
| 1 | ¥20 in 1 month | ¥22 in 1 month and 1 week | A or B | |
| 2 | ¥20 in 1 month | ¥28 in 1 month and 1 week | A or B | |
| 3 | ¥20 in 1 month | ¥34 in 1 month and 1 week | A or B | |
| 4 | ¥20 in 1 month | ¥40 in 1 month and 1 week | A or B | |
| 5 | ¥20 in 1 month | ¥46 in 1 month and 1 week | A or B | |
| 6 | ¥20 in 1 month | ¥52 in 1 month and 1 week | A or B | |
| 7 | ¥20 in 1 month | ¥58 in 1 month and 1 week | A or B | |
| 8 | ¥20 in 1 month | ¥64 in 1 month and 1 week | A or B | |
| 9 | ¥20 in 1 month | ¥70 in 1 month and 1 week | A or B | |
| 10 | ¥20 in 1 month | ¥76 in 1 month and 1 week | A or B | |

**Supplementary Figure 1**. Lottery boxes


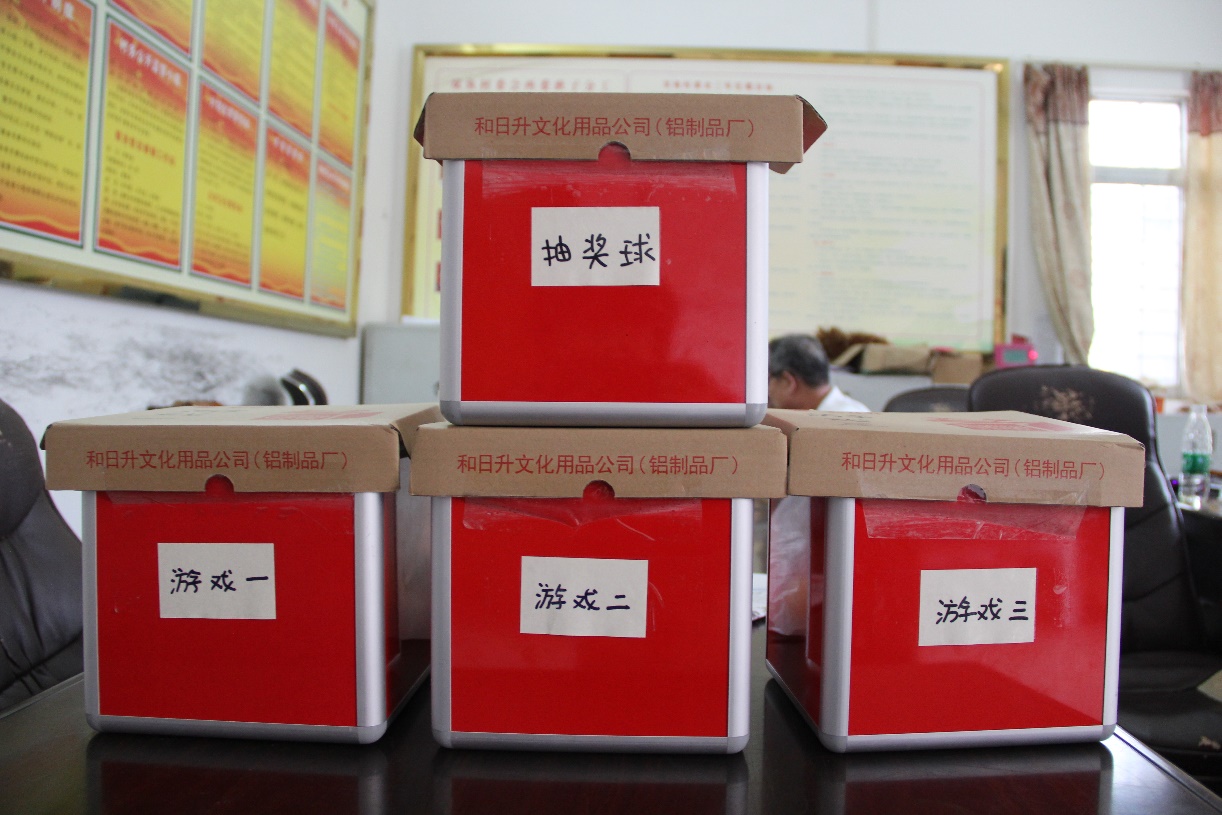


**
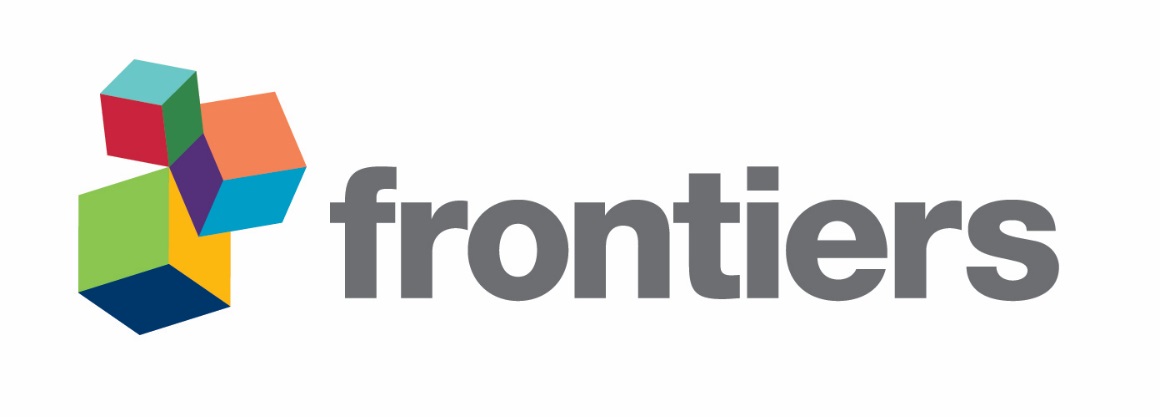
**
